# Social normative origins of the taboo gap and implications for adolescent risk for HIV infection in Zambia

**DOI:** 10.1016/j.socscimed.2022.115391

**Published:** 2022-11

**Authors:** Sophia N. Nesamoney, Iván Mejía-Guevara, Beniamino Cislaghi, Ann M. Weber, Michael T. Mbizvo, Gary L. Darmstadt

**Affiliations:** a Human Biology Program, Stanford University, Stanford, CA, USA; b Department of Medicine – Primary Care and Population Health, Stanford University School of Medicine, Stanford, California, USA; c Stanford Aging and Ethnogeriatrics (SAGE) Research Center, Stanford University School of Medicine, Stanford, California, USA; d Department of Global Health and Development, London School of Hygiene and Tropical Medicine, London, UK; e School of Public Health, University of Nevada, Reno, NV, USA; f Population Council Zambia, Lusaka, Zambia; g Department of Pediatrics, Stanford University School of Medicine, Stanford, CA, USA

**Keywords:** Sexual and reproductive health, HIV, Social norms, Gender equality, Premarital sex, Attitudes, Behaviour, Peer education, ZDHS, Zambia Demographic and Health Survey, PEPFAR, President's Emergency Plan for AIDS Relief, HIV, Human immunodeficiency virus, AIDS, Acquired immune deficiency syndrome

## Abstract

Zambian Demographic and Health Survey data reveal that increased discordance between professed attitudes and measures of behaviour regarding premarital sex among adults is strongly associated with increased risk of HIV in adolescents, particularly girls. We hypothesised that this was due to the reluctance to talk about premarital sex, resulting in a situation we call the “taboo gap” where sexual behaviour is a forbidden topic and adolescents feel unable to seek advice or sexual and reproductive health services. Our analysis revealed that the taboo gap is rooted in harmful gender norms that are perpetuated by schools, churches, cultural influences, development programmes and health systems. Challenges like food insecurity and household poverty may place girls in positions where they are vulnerable to sexual exploitation, increasing their risk of exposure to HIV. Unmarried adolescents, particularly girls, report being ridiculed when they go to reproductive health clinics, which discourages them from seeking care in the future. Strengthening peer support and parent-child interactions are important programmatic elements. We conclude that discordance serves as a novel measure and harbinger for the presence of gender norms which generated a taboo gap that impeded carseeking and increased risk for HIV among adolescents, especially girls, in Zambia. We propose that successful interventions must involve a multifaceted, gender transformative approach which engages peers and stakeholders in schools, churches, clinics, and families, particularly parents, to reduce the gendered gap in HIV risk and transmission.

## Highlights

• Premarital sex attitude-behaviour discordance is associated with risk of adolescent HIV.
• Taboo gap occurs where sexual behaviour is a forbidden topic and impedes care-seeking.
• Taboo gap was generated in schools, churches, culture, and development programmes.
• Unmarried adolescents, especially girls, risk sanction in seeking reproductive care.
• Strengthening peer support and parent-child interactions are important programmatically.

## 1 Introduction

### 1.1 Discordance in premarital sex attitudes and behaviours: a quantitative observation

Zambia has the seventh highest HIV prevalence rate among countries: 11.1% among adults aged 15–49 years, with an incidence to mortality ratio of 1.35 (UNAIDS, 2021). The HIV burden disproportionately affects females; prevalence is 14.2% among females aged 15–49 years compared to 7.5% for males of the same age (DHS Program, 2018). An analysis of 2007 Zambia Demographic and Health Survey (ZDHS) data, reported in *The Lancet* Series on Gender Equality, Norms and Health, revealed the presence of differences (or discordance) between attitudes (the way we perceive things) and behaviours (the way we act) regarding premarital sex (Weber et al., 2019). While most adults (80%) said they did not support sexual relationships prior to marriage, their behaviour was not consistent with their beliefs. Among those surveyed, 50% of adult women's and 80% of adult men's reported age of first intercourse predated their age of first union, signaling that they had engaged in sex before marriage. An adolescent girl's risk of developing HIV was found to be closely tied to the level of discordance between adult behaviours and attitudes towards premarital sex in her community. When discordance increased by 10% in a given community, the risk of HIV in adolescent females rose significantly by nearly 30%. The risk of HIV with increased discordance was also higher in boys but the relationship did not reach statistical significance.

In this paper, we seek to address several questions related to the origin, manifestations and implications of these findings: 1) Why is adult discordance in attitudes and behaviours regarding premarital sex associated with increased risk for HIV in adolescents, especially girls? 2) How do social norms lead to distinct sexual behaviours in adolescent girls versus boys? 3) What types of structures (schools, religion, etc.) create and perpetuate these norms? 4) What is the impact of these norms on care seeking behaviour in adolescent girls and boys? 5) What types of interventions have been attempted, and shown to be successful in changing harmful social norms related to premarital sex attitude-behaviour discordance among adults and consequent adolescent risk for HIV, and increasing safe sexual practices? What are the benefits and drawbacks of these approaches?

### 1.2 Social normative origins of premarital sex discordance and the “taboo gap”

Identification of a striking quantitative relationship between discordance in premarital sex attitudes and behaviours and the risk of HIV infection in adolescents, especially girls, invoked questions of why individuals' attitudes and behaviours differed, and why their discordance was associated with heightened risk for HIV infection in adolescence. Several theorists have argued that social norms – including injunctive norms which are the (mostly) unwritten rules of acceptable actions in a group – can pressure people into conforming with what they believe others expect of them (Legros and Cislaghi, 2019). However, while publicly detectable action is under strong social influence (because people know they're being watched by others), more private actions (such as sexual practices) can escape the injunctive power of the norm (Cislaghi and Heise, 2018). Clearly, when one goes secretely against the norm (e.g., having premarital sex in a place where people disapprove of that), they likely won't admit it to others, afraid of the possible social repercussions (gossiping, scolding, isolation, violence, for instance). We hypothesised that a similar system of injunctive norms is what drove the stark misalignment of people's professed attitudes and their behaviours.

When premarital sex is against the norm, individuals might hide that they are having premarital sex for fear of sanctions. The fact that they are not willing to speak about their premarital sexual practices might reduce their likelihood to seek help or services when needed. This leads to a concept that we call the “taboo gap,” where adolescents, and girls in particular, are missing out on vital services such as HIV testing, protection from pregnancy and sexually transmitted infections, notably including HIV, and abuse counseling because of the norms that exist in their communities around sexual behaviour.

We propose that similar injunctive normative taboos can create harmful situations, in this case for adolescents, by increasing their vulnerability towards HIV. We suggest that girls might be more vulnerable than boys to the consequences of the taboo gap due to double standards in how people judge boys and girls who have premarital sex (Cislaghi et al., 2021a, Cislaghi et al., 2021b). Family planning programmes that do not address similar taboo gaps might be ineffective, as suggested by analysis showing that restrictive community attitudes toward premarital sex were associated with reduced contraceptive use (Mejía-Guevara et al., 2020). In this paper, we examine this viewpoint through the synthesis of existing literature supported by descriptive analyses of Zambian survey data.

## 2 Methods

To gain insights into social origins of discordance in premarital sex attitudes and behaviours, and to understand the generation of the taboo gap, we conducted two types of analyses; quantitative ZDHS data analysis and a qualitative literature review.

### 2.1 Data analysis

We conducted descriptive quantitative analysis of four survey rounds of the ZDHS from 2001 to 2018. The four rounds of the ZDHS were conducted during the time periods 2001–02, 2007, 2013–14, and 2018. For the respective periods, women's data were obtained from individual recode files and were merged with the corresponding men's recode files. We considered the following indicators, stratified by province (1–5) and nationally (6–7): 1) discordance between attitudes and behaviours among adolescents and adults regarding sex before marriage; 2) premarital sex rates of adolescent men and women, 3) HIV infection rates of adolescent married and unmarried women; 4) child marriage (before age 18) rates; 5) percentage of adolescent men/women with multiple sexual partners (including wife/husband) during the last 12 months; 6) sex- and age-specific (adolescents, adults) HIV infection rates over time (2001, 2007, 2013, and 2018); 7) age distribution of men who are HIV positive and age distribution of their last sexual partner. Indicators 1–5 were only estimated with 2007 data and indicator 7 was estimated with 2013 and 2018 data. Adolescents were defined as ages 15 to <25 years, as defined by *The Lancet* Commission on Adolescent Health and Wellbeing (Patton et al., 2016), and adults were 25 to <50 years.

### 2.2 Literature review

A narrative literature review was conducted to gain an understanding of the cultural, regional, historical, and educational variables that may influence adult and adolescent premarital sex attitudes and behaviours in Zambia, both in the context of the HIV/AIDS epidemic of the early 2000s and in the modern-day. We searched Google Scholar using keywords such as “HIV Behavioral Discordance” and “HIV Behavioral Discordance Zambia.” However, these terms primarily yielded papers on couples with HIV-discordant status, and it appeared that the terms “behavioral discordance” or “taboo gap” had not been used in other literature. Additional searches were conducted in Google Scholar using modified terms such as “Zambia Attitudes Towards Premarital Sex”, “Premarital Sex Zambia”, “HIV Peer Education Zambia”, “Media Influences Premarital Sex Zambia,” and “Peer Influences on Premarital Sex Zambia.” Snowball searches of relevant articles were also conducted. The criteria included both observational research studies and literature reviews on the subject in English, spanning the years 1990–2020. Information extracted included quotes from survey respondents, as well as both qualitative and quantitative findings related to messages, attitudes, beliefs, and behaviours regarding sexual and family planning practices. These methods were repeated using a second database, PubMed. We developed selective narrative literature review summary tables on generation of the taboo gap in Zambia (Table S1), manifestations of the taboo gap (Table S2), and sexual and reproductive health education initiatives which inform approaches to address the tabloo gap (Table S3).

## 3 Results

Premarital sex discordance reflects a sexual double standard that puts girls at risk for HIV.

Discordance rates for men were close to 80% for both adolescents and adults at the national level in 2007, whereas those for adolescent and adult women were 59% and 43%, respectively (Table 1). The ZDHS also indicated that 16.4% of adult men and 7.8% of adolescent men reported having had multiple sexual partners during the last 12 months, while no women reported having multiple partners. These findings highlight a pattern of sexually permissive behaviour in males which contrasts with the restrictive behaviour (or reporting) exhibited by females in this population – a contradictory standard for sexual behaviour for men compared to women. These data (i.e., no women reported having multiple partners) may also reflect a reluctance among women to volunteer information about sexual activity outside a monogamous relationship with their partner.

**Table 1:** Difference by sex in the proportion of adolescents and adults who showed discordance between attitudes and behaviours regarding premarital sex (1), premarital sex rates of adolescent men and women (2), HIV rates by union status (3), child marriage rates by sex (4), and proportion of adolescent and adult men reporting multiple sexual partners (5), in Zambia and by Province, 2007

	(1) Discordance^a^: Sex Before Marriage (Proportion)				(2) Premarital Sex Rates of Adolescents^b^		(3) HIV Rates, Adolescent Women^c^		(4) Child Marriage Rates		(5) More than 1 Sexual Partner^d^ (Rates)	
	Men		Women									
	Adolescents	Adults	Adolescents	Adults	Men	Women	Not in Union	In Union	Men	Women	Adolescent Men	Adult Men
Zambia	79.5	79.8	59.1	43.1	56.6	43.8	12.3	9.4	7.0	53.6	7.8	16.4
	(77.2, 81.9)	(78.1, 81.4)	(56.2, 62)	(41.1, 45.1)	(53.8, 59.5)	(40.7, 47)	(10, 15.1)	(7.6, 11.7)	(6, 8)	(51.6, 55.6)	(6.7, 9.2)	(15, 17.8)
Province												
Central	81.3	82.4	52.3	43.4	67.9	34.8	17.2	16.0	6.4	54.9	7.3	11.5
	(75.6, 87)	(77.9, 86.9)	(43.3, 61.3)	(37, 49.8)	(58.2, 76.3)	(27.6, 42.8)	(9, 30.2)	(9.4, 25.8)	(4.2, 9.6)	(50.3, 59.4)	(4, 13.1)	(8.5, 15.4)
Copperbelt	86.7	79.9	72.0	42.1	51.8	35.5	13.8	11.9	7.3	50.7	6.6	9.4
	(81.7, 91.8)	(75.3, 84.5)	(63.1, 81)	(36.7, 47.5)	(45.2, 58.3)	(29.3, 42.2)	(7.2, 25.1)	(7.1, 19.1)	(4.3, 12.1)	(43.7, 57.7)	(4.3, 9.9)	(6.7, 13)
Eastern	75.7	84.0	50.0	29.5	59.9	43.3	7.7	2.1	10.4	62.2	9.1	24.2
	(68.6, 82.8)	(79.5, 88.5)	(41.8, 58.2)	(24, 35.1)	(52.1, 67.3)	(35.1, 52)	(4.2, 13.6)	(0.7, 6.3)	(8, 13.3)	(58.1, 66.1)	(5.7, 14.3)	(20.8, 28)
Luapula	86.1	89.2	48.5	45.6	54.1	41.0	12.7	10.2	8.3	63.9	5.0	9.0
	(79.5, 92.7)	(86.2, 92.1)	(39.7, 57.3)	(39.2, 52)	(45.2, 62.7)	(32.1, 50.7)	(5.6, 26.6)	(5.1, 19.2)	(5.6, 12.1)	(58.6, 68.9)	(2.5, 9.9)	(6.2, 12.9)
Lusaka	87.2	86.7	66.5	49.8	51.7	40.2	13.2	18.6	4.9	44.1	8.3	19.2
	(82.7, 91.7)	(84, 89.4)	(58.6, 74.4)	(44.8, 54.9)	(44.4, 58.9)	(32.2, 48.8)	(8.8, 19.3)	(10.8, 30.2)	(3.1, 7.5)	(37.5, 50.8)	(5.5, 12.3)	(16.2, 22.6)
Northern	69.4	57.9	38.3	19.2	46.5	35.8	12.7	2.7	6.1	58.0	3.6	8.5
	(60.2, 78.6)	(51.8, 64.1)	(30.6, 46)	(15, 23.4)	(38.3, 54.9)	(25.7, 47.3)	(7.2, 21.4)	(0.8, 8.5)	(4.2, 8.8)	(53.4, 62.5)	(1.9, 6.8)	(5.9, 12.1)
North-Western	66.9	70.5	63.1	56.4	71.9	60.1	8.5	3.6	9.7	61.7	8.5	24.0
	(59.6, 74.1)	(63.5, 77.6)	(58.3, 67.9)	(50.2, 62.7)	(63.1, 79.2)	(51.6, 68)	(4.5, 15.4)	(1.6, 8)	(7, 13.3)	(54.8, 68.2)	(5.6, 12.6)	(18.5, 30.5)
Southern	80.1	82.9	70.4	56.2	58.3	54.7	12.0	13.7	5.6	48.5	12.1	27.8
	(74.6, 85.6)	(78.6, 87.3)	(63.4, 77.4)	(50, 62.3)	(52, 64.4)	(47.1, 62.1)	(6.5, 21.2)	(8.8, 20.7)	(3.7, 8.3)	(43.7, 53.3)	(8.8, 16.4)	(23.3, 32.9)
Western	68.9	82.8	66.7	66.8	79.5	76.6	12.5	10.0	3.7	39.6	13.3	20.3
	(60.1, 77.6)	(77.7, 87.9)	(58.3, 75.1)	(61.9, 71.7)	(71.2, 85.8)	(67.7, 83.6)	(7.4, 20.4)	(5.3, 18.1)	(2, 6.6)	(32.5, 47.1)	(9.4, 18.6)	(15.7, 25.8)

**Source:** Authors' data from the Zambia Demographic and Health Survey (ZDHS) 2007.

Notes: The values in all columns correspond to calculated rates, and their 95% confidence intervals in parentheses.

a Discordance refers to the mismatch between individuals' reported attitudes and actual behaviours towards premarital sex (e.g., an adolescent who believes she should wait for sex until marriage, but she engages in premarital sex anyway). Attitude was derived from the ZDHS 2007 question: “Young men should wait for sex until marriage.” Behaviour was derived from responses to questions on age at first sex and age at first union; if age at first sex predated age at union, then premarital sex behaviour was assumed. The column shows the weighted proportion of those mismatches at the national or regional levels.

b Premarital sex rates were approximated from reports of never in union men and women aged 15–24 who ever had sex (sexually active).

c Here we only considered sexually active adolescent women.

d No women reported having more than one sexual partner. There data refer only to men.

Despite the higher rates of premarital sex and multiple partners reported by males, females have had consistently higher rates of HIV infection than their male counterparts over the past two decades, particularly during adolescence (Fig. 1). We hypothesised that the answer to this paradoxical finding lies in understanding social norms regarding adolescent sexual behaviour, and how the taboo gap in accessing services is generated and manifested in Zambian communities. We find that although fewer adolescent girls are having premarital sex than boys (Table 1), the types of relationships they engage in may lead to a greater risk of exposure to HIV, as discussed below.

**Fig. 1:**
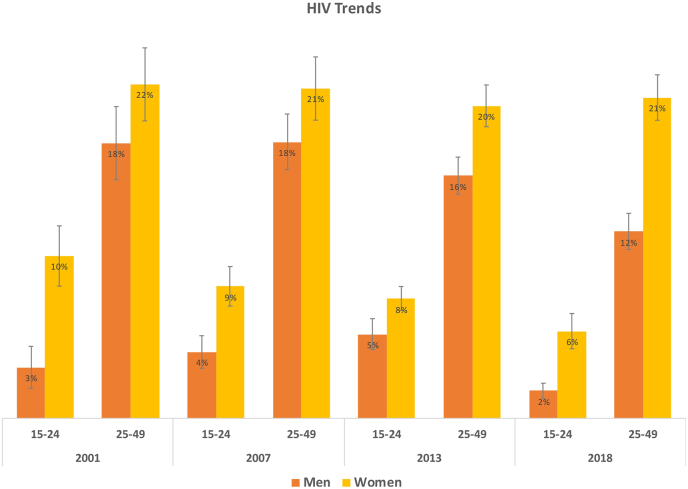
Trends in HIV infection rates in Zambia for adolescent (aged 15–24) and adult (25–49) men and women from 2001 to 2018.

**Source:** Authors’ analysis of ZDHS from 2001-02, 2007, 2013-14, and 2018." and tag "Vertical lines indicate 95% confidence intervals (CI), calculated using Taylor-linearised standard errors to account for the ZDHS complex survey design. However, since ZDHS 2001-02 HIV data cannot be linked to individual or men’s recode files that include survey design variables, CI for this wave were calculated assuming a simple random design (i.e., CIs from 2001-02 did not account for variation due to the multi-stage complex survey design).

### 3.1 **Generation of adolescent sexual norms and the taboo gap** (Table S1)

Zambia is considered a patriarchal society, ranking 45th in the World Economic Forum Gender Gap Index (Crotti et al., 2019) and 137th in the Gender Inequality Index (United Nations Development Programme, 2022). Gender norms (social norms that specifically apply to people because of their gender) are generated and upheld through influences in families, communities, institutions, structures and policies, reflecting the patriarchal context in which they are situated (Heise et al., 2019) (Hay et al., 2019). People conform with collective norms even when they, individually, believe these norms to be harmful (Merdes, 2017). Among the social forces that create, reinforce and socialise people into complying with norms, educational agencies play a key role. Non-compliance with norms may generate feelings of shame, anxiety, and fear of incurring social sanctions or divine punishments. Similar fears can become vehicles of (un)conscious social control at school (e.g., bad markings from teachers, stigma from peers, or bullying) and embodied in religious precepts (think of sins and commandments, hell and paradise, confession and penitence) and development interventions (where, as many have observed, the whole system of cooperation demands abiding to neoliberal norms of the Global North) (Ferguson, 2006). In this section, we specifically look at the role that these three institutions (schools, churches, and international aid organisations) have played in generating, contributing to, or accelerating the formation of adolescent sexual social norms, the taboo gap, and their effects.

#### 3.1.1 Influence of schools

Beginning in 1981, HIV/AIDS began to spread rapidly through the US, and by 2000 it was the leading cause of death among young males (Bendavid, 2016). On the African continent, while HIV/AIDS was not the largest burden of disease, it rose sharply in prevalence, particularly in sub-Saharan Africa, from around 600,000 cases in 1981 to 20 million in 2000. The US attempted to apply their own proposed solutions to tackle Africa's HIV epidemic under President Bush's *President*'*s Emergency Plan for AIDS Relief* (PEPFAR) (Bendavid, 2016). As a part of PEPFAR, the US spent $1.4 billion from 2004 to 2016 to implement abstinence-promoting programmes across schools in Africa, including in Zambia (Doucleff, 2016). Sex education programmes that received PEPFAR funding were required to allocate at least 33% of their budget towards promoting abstinence (Gordon and Mwale, 2006). Schools could only promote condoms for use by prostitutes and schools were forbidden from teaching middle-level students (age 10–14 years) about condoms (Gordon and Mwale, 2006).

Children who came of age in the early 2000s were denied access in schools to knowledge about safe sex and contraceptive use. The association of condoms with prostitution created stigma about contraception, associating condom use with promiscuity (Mwale, 2012). Some students also reported being taught that contraceptive use led to cancer or long-term infertility (Silumbwe et al., 2018).

Importantly, Zambian teachers have reported feeling uncomfortable while teaching sexuality education using material that was provided to them through development aid-supported programmes (Zulu et al., 2019). One common complaint was that the material teachers were given was not culturally competent (Zulu et al., 2019). Teachers in a study by Zulu et al. (2019) described feeling that the material they were supposed to teach their students was written for a Western environment rather than for their own community. One teacher said that most of her students were not fluent in English, so she had to translate the words into their local language, which sometimes came across as vulgar and disrespectful. Teachers mentioned feeling embarrassed about teaching sexuality education, with one teacher saying that she closes her eyes when she mentions sex organs. Another teacher said it was very difficult to balance her role as a parent and an educator, as she would not want her own child to be learning these topics in school. This was echoed by another teacher in Zulu's study, who said that when she tried to teach sex education, the parents from the community stormed the principal's office and complained (Zulu et al., 2019).

Ultimately, norms regarding premarital sex were shaped in the educational sector through messages conveyed through the classroom content provided (or forbidden). The lack of support and training to teachers to teach sex education well further impeded the quality of sexuality education. The messages that were conveyed in schools could have contributed to generating or reinforcing the taboo gap, where honest and open discussions about sex were prohibited and sanctioned. Linking contraceptive use with promiscuity may have had lasting effects in stigmatising condom use. Furthermore, the association of condoms with prostitutes, who in most societies are typically women, seemed to imply that females are to be blamed for the spread of HIV. PEPFAR has been one of the most successful programs in tackling HIV/AIDS, having saved an estimated 20 million lives by initiating a supply chain of more affordable antiretroviral therapies and reducing the rates of HIV/AIDS worldwide (Bendavid, 2016) (United States Department of State, 2020). It is also important to acknowledge, however, that some of the PEPFAR programming appears to have contributed to restrictive gender norms about premarital sex and the taboo nature of the topic that continue to be potent across Zambian society today.

#### 3.1.2 Influence of churches

In Zambia, approximately 95% of the population practice Christianity (United States Department of State, Office of International Religious Freedom, 2019). Despite most Zambian Christian churches promoting abstinence before marriage, churchgoers’ actions do not necessarily follow church teachings (Heslop and Banda, 2018). In a study conducted in the Chipata district, several participants admitted that, despite engaging in sex before they were married, they often pretended they did not (Mwale, 2012). One participant reflected on how this was the case for younger people: “[Our] church says that when you have sex before marriage you will be judged as a sinner, but they [young adults] do it anyway.” If adolescents want to have sex but are afraid to be called a sinner and suffer sanctions, they may (and, our findings suggest, most likely will) engage in sexual behaviours secretly. This may create barriers in asking for help or advice from others in the community – the taboo gap – especially about topics concerning sexual health, pregnancy prevention, and HIV transmission.

#### 3.1.3 Cultural influences in the community

In addition to schools and churches, norms are taught, reinforced, and, in some cases complicated by Zambian cultural beliefs. One example of this is seen in initiation ceremonies which are traditionally performed for both adolescent girls and boys in Zambian culture and signal the passage into adulthood (Heslop and Banda, 2018).

Female initiation ceremonies usually occur at the onset of menstruation and emphasise the young woman's role as a homemaker (Heslop and Banda, 2018). Girls are taught skills for how to manage a home, as well as how to sexually please their future husband. Adolescent girls from a village in Eastern Zambia reported being given *lunkhanko*, a traditional medicine which serves as a reminder to teen girls to remain a virgin. These girls said they were told that the medicine would kill them if they engaged in sex before marriage. Thus, the cultural lessons that girls receive are concordant with the teachings of the church and school, perhaps contributing to their relatively high concordance between premarital sex attitudes and behaviours.

Boys, however, receive a contrasting cultural message. During their initiation ceremonies, they are told that frequency of engaging in sex is a defining characteristic of manhood (Heslop and Banda, 2018). Adolescent males from the same village in Eastern Zambia reported being given herbs to enhance their immediate sexual desire (Heslop and Banda, 2018). Unlike girls, boys are taught that their ability to have sex many times is important for proving their masculinity. This lesson appears to be in direct conflict with what they have been taught in schools and churches and could contribute to disproportionately higher rates of discordance between boys' premarital sex attitudes and behaviours. Although boys and men know that their church and school advocate that they wait until marriage, sanctions are less apparent than for girls and conversely, cultural pressures surrounding masculinity appear to create a strong pull towards behavioral dissonance from attitudes professed publicly. A study by Sommer et al. (2015) in neighboring Tanzania showed that boys used puberty as a justification for their sexual desires and behaviour. One boy discussed a sexual encounter he had: “I remember it's not my mistake because it's puberty. That's why I did such a thing. It's a normal thing for a boy in puberty.” According to Sommer et al. (2015), male puberty is seen as a collection of “all-powerful sexual emotions that cannot be resisted,” which then excuses male pre-marital sexual behaviour and promiscuity, and possibly even excusing more sexually violent behaviour.

Boys in Zambia, like in many other parts of the world, are also trained from a young age to be “providers,” which can also greatly influence how they practice sex and with whom (Sommer et al., 2015). Oftentimes, boys will leverage this principle and exchange gifts such as food, alcohol, or even money with girls at school in order to encourage them to have sex. As is discussed in the next section, this may cause girls to feel like they owe boys sex if they accept these gifts.

### 3.2 **Adolescent girls’ vulnerability to the taboo gap with decreased care seeking and heightened risk for HIV infection** (Table S2)

A variety of social and economic factors appear to generate power imbalance in pre-marital sexual relationships, which may become an important way through which adolescent girls’ risks of acquiring an HIV infection is increased. We hypothesise that high rates of child marriage and/or transactional sex may contribute to increased risk for HIV acquisition among adolescent girls relative to adolescent boys, although further research is needed to examine these mechanisms and interactions among them.

#### 3.2.1 Child marriage

We explore the potential role of child marriage – a human rights violation (United Nations Human Rights, 2022) – and socioeconomic factors and social norms associated with this illegal practice in contributing to adolescent girls’ heightened risk of HIV infection. In Zambia, the legal age of marriage is 21 years (Kauseni, 2018). Although child marriage is a violation of Zambian constitutional law, the prevalence of child marriage has remained consistently high in Zambia since the early 2000s, with rates of 60% in the Eastern Province and 48% in the Northern Province (Kauseni, 2018). The practice is less common in urban settings – the capital of Lusaka has a lower rate of around 44% (Table 1). The ZDHS shows that females were much more likely to experience child marriage than males. In Zambia in 2007, only 7% of men were married before their 18th birthday, compared to 54% of women (Table 1).

Community pressure is an important driver of child marriage, as families who do not practice child marriage for their girl may be ostracised in some settings (Kauseni, 2018). Parents also may try to marry their daughters as teens or preteens to avoid them engaging in premarital sex. Moreover, many parents in Zambia believe that early marriage can protect their daughters from HIV, as they hope that their daughter's husband will only have one sexual partner (Kauseni, 2018). In many cases, child marriage is driven by socioeconomic factors. A study by Kauseni (2018) showed that poverty can increase the rates of child marriage. When a girl is married, her parents will no longer need to provide for her financially, which reduces her burden on them. In addition, the bride's family may receive a dowry or lobola, typically in the form of money or livestock, as a pre-wedding sign of commitment on the part of the groom (Kauseni, 2018).

Data from Zambia is mixed on the association of child marriage with risk of HIV infection. When considering all adolescent girls in Zambia, both those who are sexually active and those who are not, the 2007 ZDHS indicated that HIV infection is higher for married (9.5%) than for unmarried (6.8%) girls. These data are consistent with findings from Glynn et al. (2001), who reported that HIV prevalence was 10% higher in married (27%) than unmarried (17%) adolescent girls in Ndola, Zambia. In contrast, considering only sexually active adolescent girls in the 2007 ZDHS survey, married girls were less likely to have contracted HIV than those who were unmarried (9.4 vs. 12.3%) (Table 1), for reasons we explore in part in the next section on transactional sex. While some girls may want to marry early, for others, child marriage may force them into a sexual relationship earlier than they otherwise would choose. Imbalanced gender and age power dynamics and lack of decision-making power^19^ within the marriage may also make it difficult for the married girl to negotiate safe sex practices, particularly condom use, or to ask her partner to be tested for HIV, thus potentially increasing her exposure to HIV, especially if the husband is having sex with other people (Kauseni, 2018). More research is needed, however, to more fully understand the effects of child marriage on HIV risk.

#### 3.2.2 Intergenerational and transactional sex

**Fig. 2:**
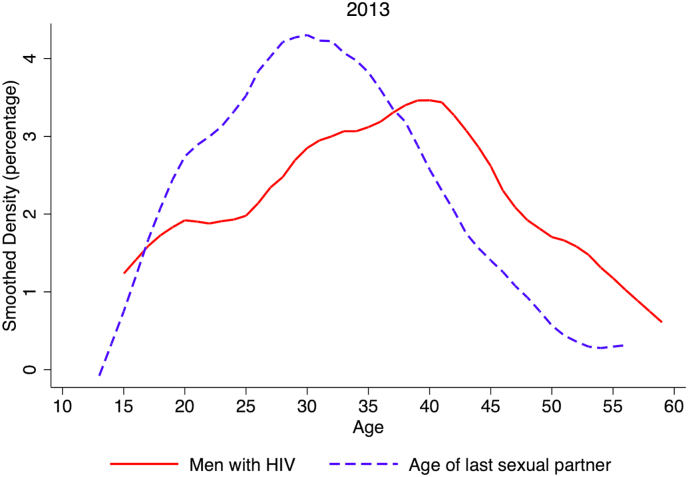
Age distribution of men who are HIV positive and age distribution of their last sexual partner, Zambia Demographic and Health Surveys, 2013

**Source:** Authors’ analysis of ZDHS from 2013. and tag "The plots represent smoothed density functions, reported as percentages in the Y-axis (e.g., in the solid red line, the value at age 30 indicates that ∼3% of HIV positive men were 30 years old in 2013).

Another potentially important socioeconomic factor related to girls’ increased risk for HIV infection is the common practice of intergenerational sex, or transactional sex which often occurs between unmarried younger women with older men*.* Data from the ZDHS from 2013 to 2018 indicates that most HIV-positive adult men across a wide age-range regularly engaged in sex with women much younger than themselves (Fig. 2: 2013, Fig. 1S: 2018). It is important to note that many of these HIV-positive men were coming of age during the initial spike of HIV/AIDS in the 1990s and early 2000s. These men may have acquired HIV infection at that time, and years later, passed it to younger female sexual partners. This important phenomenon of intergenerational HIV transmission was first described in South Africa where recent spikes in HIV infection have been seen among adolescent girls, and may also be an important contributing factor in Zambia, although further research is needed (Conway-Smith, 2015).

It is important to consider the reasons why girls could benefit themselves or their families from their sexual relationship with an older man. Some adolescent girls may turn to transactional sex with older men in their community for short-term financial stability or freedom. However, while a desire to buy commodities may be a factor, a study conducted in 2019 found that the main driver for these relationships was food insecurity in the girls' families (Wamoyi et al., 2010) (Austrian et al., 2019). Oxfam in 2008 found that in households that had been affected by past HIV/AIDS epidemics, there were gender differences in access to food and when families face economic or food-related hardships, girls are the ones who suffer the most (Aikman et al., 2008). Girls are often required to take on extra duties in the home, even if this means missing out on school and the future opportunities associated with education. “There is a lot of hunger in the homes, which means that at times the child goes without anything to eat all day,” said one parent interviewed by Austrian et al. (2019). “When a man who is offering her K10 (=0.80 USD) for her company approaches her, she will end up pregnant”. A sixteen-year-old girl from the study said she felt guilt at not offering sex: “When he asks you for sex, you never refuse because you will think of his money that you have already spent” (Austrian et al., 2019). It is estimated that 38% of Zambian girls have engaged in sex for money (Luke and Kurz, 2002). The exchange of sex for food or money often means that the girls have little power in negotiating safe sex, hence the frequency of pregnancies associated with these relationships. A large age gap in transactional sex means that there is increased likelihood that the partner in transactional sex may already be HIV-positive, especially if he is engaging in sex with multiple women. Thus, as in child marriage, girls in transactional relationships may not be able to negotiate condom use and may therefore face a higher risk of HIV exposure. The link between transactional sex and pregnancy is also crucial, as girls engaging in transactional sex were 37% more likely to become pregnant than those who were not engaging in these types of relationships, and rates of pregnancy increased as the male partner's age increased (Austrian et al., 2019). If girls in these relationships are becoming pregnant at such high rates, one can assume that unprotected sex is common. Teen pregnancy decreases girls' education and employment opportunities, and further increases their vulnerability (Shakya et al., 2020).

Rates of HIV exposure may be further accentuated in a normative environment where girls’ older partner feels that he needs to assert his masculinity by having multiple extramarital partners. As this behaviour could reflect common cultural norms, these men may not feel that what they are doing is “wrong.” On the other hand, girls are told to avoid promiscuity at all costs, so they may be more likely to feel guilty for engaging in the relationship. Therefore, if the girl or her partner tests positive for HIV, both the girl and her partner are likely to place the blame on her. This may cause her to conceal her behaviour, potentially preventing her – moreso than her male partner – from seeking important health care, creating a taboo gap which could enhance her risk for HIV.

Even in relationships without a large age difference, girls are still at disadvantage when it comes to negotiating power. In a 2013 survey, Heslop and Banda (2018) found that girls aged 16–29 years in the Zambian Eastern Province were told to never initiate sex and to initially refuse, to prevent being seen as promiscuous. Consent was largely seen as a prize for boys to win, not something that girls could actually deny. “A girl will say no even if she wants sex – she can't seem too eager or she will be seen as a prostitute,” said one male who participated in Heslop's survey. “But [boys] can tell by the way she talks and her actions that she's really interested”. Several females in the study said that this assumption made it difficult for them to communicate a firm refusal of sex and made condom use nearly impossible, as “the game was that males were supposed to overpower females.”

It is also important to highlight that although ZDHS data shows higher rates of discordance in males than females of all age groups, the rates of discordance in current adolescent females are much higher than the discordance of generations before them (Table 1). This may signal a shift in behaviour, where girls are engaging in premarital sex at higher rates while still being caught up in the taboo gap and thus are facing higher levels of potential exposure to HIV while not seeking protective health care services.

#### 3.2.3 Gendered health systems

Adolescent girls may face pushback within their health systems if they seek preventative care (Hay et al., 2019). In Zambia, until 2005, women were not allowed to obtain contraceptives unless they had consent from their husband (Belohlav and Karra, 2013). Therefore, in the present day, stigmas may still exist that prevent adult women from obtaining sexual and reproductive health services. Of greater importance is the fact that currently in Zambia, adolescent girls under the age of 16 must obtain parental or spousal consent in order to receive sexual and reproductive health services (Government of Zambia Human Rights Commission, 2017). This means that there is a legal barrier that prevents most adolescent girls from consenting to preventative care for HIV, contraception, or gynaecologic exams. As a result, when an adolescent girl tries to be proactive about her own sexual health, she may be turned away.

Over the past decade, researchers have found that this refusal of care is a very real issue that unmarried adolescent girls and boys are facing (Silumbwe et al., 2018). Adolescents in these studies who tried to seek care reported being ridiculed and scolded for seeking condoms. “Youths in most cases, when they go to the health facility to access family planning, the attitudes of caregivers send them away,” said a survey respondent in a study by Silumbwe et al., “especially when they are scolded to say, ‘you're still in school, you're supposed to concentrate on books and not come in for family planning’” (Silumbwe et al., 2018). Another survey found that many adolescents who visited these clinics were concerned at the lack of privacy and did not trust that the health workers would maintain their confidentiality (Mmari and Magnani, 2003) (Feldman et al., 1997) (Isaksen et al., 2022).

The failures of the reproductive health clinics extend beyond family planning, particularly for adult women who seek information about sexual health or who are interested in learning about how to stop the spread of HIV. This challenge is accentuated for women who engage in transactional sex, as they may be denied contraception unless they are with a male partner who consents (Mukuka and Slonim-Nevo, 2006). Moreover, methods that women have bodily autonomy over, such as injectables, intrauterine devices or implants, protect against pregnancy, not HIV (Grabbe et al., 2009). To be protected against HIV, women must rely on their male partner using condoms, which robs them of the autonomy to protect themselves from disease (Grabbe et al., 2009).

The dismissal and shaming exhibited by service providers may widen the taboo gap, making girls feel embarrassed about their sexual behaviour and fearful of sanctions. Negative experiences at these clinics may make them less likely to prioritise their sexual health care in the future. As a result, girls may contract HIV and not know it, increasing the spread of the disease. Furthermore, they may be less likely to seek help from elders in the community if they feel unsafe in their intimate relationships, as they may worry that they will be shamed for engaging in sex on one hand, and on the other hand, may be subjected to violence from their partner who may perceive their seeking help as a threat (Weber et al., 2019).

Finally, we acknowledge that there are many other reasons, besides the normative mechanisms that we have explored here, which may explain differences in HIV rates between adolescent girls and boys. For example, biological mechanisms may play a role (Mahathir, 1997) (Abbai et al., 2016). Also, increased HIV rates in females might be because they have more access to testing and health services, such as prenatal care; indeed, there are a number of other factors that could be explored, many of which may also have normative influences (Birdthistle et al., 2019) (Cornell et al., 2012) (Cornell et al., 2011) (Vandormael et al., 2019).

### 3.3 **Current gender transformational initiatives for shaping adolescent sexual norms and combatting the taboo gap** (Table S3)

To address the taboo gap, formal health education could offer an important piece of the solution. Formal education can greatly influence behaviour, so it is crucial that schools feel prepared to provide students with accurate and culturally sensitive information about sex and sexual health. Several studies have found that sex education can be a subject that teachers, who are often much older than their students, feel uncomfortable discussing in the classroom (Mwale, 2012). These teachers may feel that their students will not take them seriously or that they themselves are ill-equipped to provide guidance to students. Due to the potentially large age gap between teachers and students, students may also feel uncomfortable discussing sexual health openly with their teachers.

An important opportunity therefore presents itself for students themselves to become peer health educators who provide their peers with resources about sexual and reproductive health. They can also serve as a safe resource where students can seek help if they find themselves navigating exploitative or otherwise harmful relationships. Svenson et al. found that most students in Zambia viewed peer education favourably and wished to see it implemented more in their schools (Svenson et al., 2008). They also found that exposure to peer health education was correlated with increased condom use and a potential decrease in the number of sexual partners. In addition, peer health educators played an important role in referring students to clinics, particularly those who were at-risk for sexually transmitted infections or pregnancy or had other sexual health needs. This formed an important bridge between schools and local clinics, allowing students to feel supported and empowered in addressing their sexual and reproductive health needs.

Hughes-d’Aeth found that an effective way to approach peer health education was to use a multi-sectoral approach, aligning it with interventions like psychological counseling opportunities and with income generation programmes (Hughes-d’Aeth, 2002). They found that it was crucial to consider the role of poverty, urbanisation, sanitation, access to healthcare, and gender when implementing peer education programmes, given the role of these factors in vulnerability to HIV infection.

A study by Butts et al. (2017) found that adolescents believed it was important to incorporate multiple types of education (such as television programs, pamphlets, plays) into peer health education for the teachings to be effective. They also mentioned that it was important for peer educators to be trained on how to support students who are experiencing intimate partner violence or rape.

One challenge that must be addressed is ensuring that interventions are transforming behaviours, not only attitudes, thus reducing attitude-behaviour discordance and high-risk behaviours. For example, using a quasi-experimental design, Denison et al. implemented a peer sexual health education programme for students in grades 8–9 across 15 Zambian schools and evaluated students’ knowledge about HIV, sexual health, and reproductive anatomy (Denison et al., 2012). When compared to students from the 15 control schools in 2012 in which no peer sexual health education intervention was introduced, students in the experimental schools were 1.6 times as likely to answer 19 or more out of 24 sexual health questions correctly. These students also exhibited greater confidence in their ability to refuse sex and were more likely to list condoms as an important part of safe sex. However, reminiscent of the attitude-behaviour discordance seen in the ZDHS, the researchers found inconsistent evidence for whether changes in the sexual behaviour of the experimental students were aligned with their expressed opinions and knowledge about sex. While students in the schools which received the programme expressed greater self-efficacy to refuse unwanted sex and access condoms, there was no difference in the percentage of students in the two groups who had engaged in sex and most experimental school students did not use condoms. However, intervention students were more likely not to have had sex in the previous year and to have had only one sex partner ever.

While peer education is a promising approach, improving the parent-child dynamic may also show positive results. A study by Wamoyi et al. (2014) in sub-Saharan Africa showed that improving parent-child communication about safe sex could reduce the risk of adolescents participating in unsafe sex practices. Parents play a large role in the way that children see the world, so if parents are willing to engage in open and honest questions about sexual health, then their children may be able to seek help, advice, or preventative care when needed. Parents also play an important role in deciding what material is taught in schools, so ensuring that parents understand the importance of sex education and feel included in discussions about sexual health is crucial.

A study by Zulu et al. (2019) supports this idea as well. After honest discussions about gender, sexuality, and sexual health, parents reported that their children were hiding less from them and that their children had started to trust them more. Many of these parents described feeling restricted by cultural norms around what they should and should not discuss with their children, so they felt empowered when they were invited to freely engage in these types of conversations. Programmes which engage parents in talking about sexual and reproductive health are particularly important in light of the finding that it was adults’ discordance in premarital sex attitudes and behaviours that was found to be associated with adolescent risk for HIV. Notably, some parents said that these conversations shifted their priorities towards their female children (Zulu et al., 2019). One parent explained that she realised that she should prioritise the education of her daughter rather than sending her to do house chores. “I did not know that children had rights and responsibilities,” the parent said. “When I learned about the rights of children, we discussed this with my child and I gave her time and space to study.” Another parent explained that these conversations made her wait to marry her daughter off until after her daughter had completed school. “Our tradition always taught us that the pride of a woman is marriage … I learned that the girl child can complete education and still be of great contribution to the family and the community at large … The idea of marrying my daughter off is a nightmare now.” However, Zulu et al. (2019) also found that parents may not agree on what type of information is ethical to discuss with their children. Some parents in the study believed that they should not talk about condoms with their children, although they were open to discussing other topics.

Finally, it is also important for programmes to recognise the “Sugar Daddy” phenomenon – where girls and women engage in sexual relationships with older men in exchange for money or gifts – and address the complex sexual relationships that adolescent girls may be navigating today (Conway-Smith, 2015). Although there have been limited efforts to combat the intergenerational spread of HIV in Zambia, public health efforts in South Africa have been promising (Conway-Smith, 2015). Aaron Motsoaledi, the former South African Minster of Health, declared “Sugar Daddies must fall” following the implementation of economic programmes that would help young women receive higher education and better job opportunities (Conway-Smith, 2015). These efforts, the government believed, could help to alleviate some of the financial burdens that increase young women's vulnerability to being vicitimised in exploitative sexual relationships. A study of interventions across sub-Saharan Africa found that economic programmes such as the “Girls Power Initiative”, which trained women in employable skills, reduced the rates of young women engaging in transactional sex (Wamoyi et al., 2014). A study by Bermudez et al. (2021) highlighted the importance of financial freedom in decision-making,^40^ noting that adolescence is a unique time financially, as individuals are beginning to enter the realm where they may no longer be financially supported by their parents. This brings forth both uncertainty and freedom, as teens with their own money can purchase items without parental permission. Bermudez et al. found that teen girls who had more economic stability were able to avoid high-risk sexual behaviours, which speaks to the importance of finances in the spread of HIV. By addressing the complex financial and job-related considerations of girls facing these types of relationships, programmes can help them feel understood and supported instead of scolded or judged.

## 4 Discussion

### 4.1 Explanatory hypothesis for the taboo gap

**Fig. 3:**
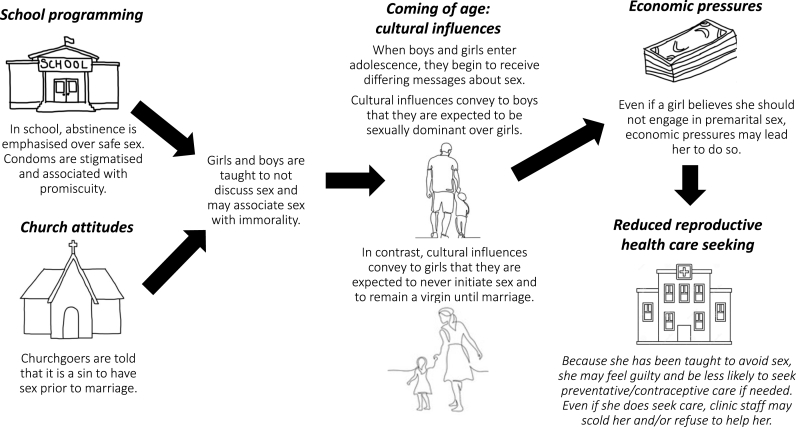
Explanatory hypothesis for formation of the taboo gap in seeking sexual and reproductive health services related to premarital sex.

The above analyses led us to develop an explanatory hypothesis, informed by the theoretical literature on gender norms presented earlier, for how the premarital sex taboo gap forms and how it leads to increased adolescent girls' risk of contracting HIV (Fig. 3). Before puberty, boys and girls tend to be socialised within similar sets of restrictive norms proscribing premarital sex (Heslop and Banda, 2018). As children reach puberty and begin to manifest adolescent body attributes, boys and girls begin to be socialised in different directions, as they are channeled within existing gender roles for men and women. Child marriage may be a community norm and a way for parents to secure their daughter's financial stability and protect her from HIV, while also guarding against the shame of an out-of-wedlock pregnancy (Kauseni, 2018). Thus, unmarried adolescent girls are policed into sexual restraint and exposed to a consonant system of norms sanctioning them for having premarital sex (e.g. Cislaghi et al., 2020) (McCleary-Sills et al., 2013). Boys, instead, might get exposed to a cultural norm of proving their emerging manhood through sexual prowess, as their masculinity is tied to their ability to have sex frequently and their dominance over girls (Heslop and Banda, 2018) (Sommer et al., 2015). While these boys might still carry internalised norms against premarital sex, they are also confronted with norms prescribing sexual activity (Heslop and Banda, 2018). Interestingly, this contradictory bundle of norms (prescribing both sexual restrain and prowess) is reminiscent of those that American teenage girls have to navigate (Mollborn and Sennott, 2015). As time passes, girls as well might experience sex: because they desire it; in exchange for gifts, money or food; or because they enter in a partnership with the hope it might lead to marriage, to cite a few possibilities. Due to the greater stigma associated with girls' sexual activity, however, they might be less prone than boys to seek help and advice. When they do seek care, however, girls may be denied access to sexual and reproductive health services unless they come with a spouse, parent or guardian. Moreover, they may face sanctions if it becomes known that they are sexually active, and may be likened to a prostitute if they seek to obtain condoms. The taboo gap prevents adolescents – both girls and boys – from seeking care and also prevents health workers from responding appropriately. Thus, we believe that several factors contribute to the creation of the taboo gap, and the diverse ways in which it affects girls' and boys' exposure to HIV, including (1) the differential unfolding system of norms for boys and girls, paired with (2) the fact that girls might experience child marriage and transactional sex, as well as (3) a biological predisposition to contract HIV, and (4) the lingering norms within the health system that might refuse the provision of services to single or underage girls who may fear that exposure of their premarital sex practices could lead to sanctioning. Finally, we note the need for caution in interpretation of our explanatory hypothesis. While data from the ZDHS are gender binary, and are often used to examine heterosexual relationships, in our case the nature of boys' and girls' relationships is unconsequential. Because of the system of norms allowing boys to boast about their sexual encounters (that would, nonetheless, happen secretely), we hold that they might have better access to services than girls independently of their sexual orientation. Despite this possible advantage, boys who have sex with boys would do so paying the excruciating psychological cost of having to lie about their sexual orientation to survive in a restrictive and potentially violent heteronormative social order (Müller et al., 2018).

### 4.2 Limitations

There are several limitations to this research. While our literature review identified a solid body of qualitative evidence that informed our research questions, our review was not systematic and could have included more search terms related to social, gender and cultural norms. This research focuses more on heterosexual relationships, which means that we were not able to address the burden of HIV on LGBTQ + adolescents and adults in depth. Furthermore, while we have highlighted the importance of involving parents in discussions about sexual health and involving teachers in designing culturally appropriate sex education curriculums, the details of how these initiatives would be undertaken need to be explored further. Finally, the quantitative data collection occurred from 2007 to 2008, so it is important to acknowledge that the rates of premarital sex and attitudes towards premarital sex may have changed since then.

## 5 Conclusions

Inculcation of gender norms in homes, churches, schools, and cultural events can create a taboo gap wherein sex prior to marriage becomes a forbidden topic that leads to reduced access to sexual and reproductive health services (Fig. 3). This taboo is systematically reinforced and sanctioned, including by parents, peers, teachers, communities, development programmes and health systems, making it difficult for young people to learn how to practice sex safely (Kågesten et al., 2016) (Hay et al., 2019). Adolescents’ concerns about patient privacy and being ridiculed or turned away when seeking care highlights that it is not enough to ensure that hospitals and clinics are fully stocked with contraceptives. We acknowledge that there are many other issues related to provision of sexual and reproductive health in addition to the normative factors we focus on here, however, it is important to note that a failure to address normative issues, for example by focusing only on supply-side factors, may render sexual and reproductive health programmes insufficient to meet the needs of adolescents. Even if reproductive clinics have an adequate supply of condoms, harmful norms and fear of judgement may generate a taboo gap – belied by discordance between premarital sex attitudes and behaviour – that inhibits adolescents from seeking information and care that could protect them from HIV. It is important to make sure that all levels of health care providers are comfortable addressing the needs of teens who seek sexual and reproductive health care, and that they do not approach their adolescent patients with judgement.

Higher rates of discordance between their the attitudes and pre-marital sexual behaviours of boys than girls are a manisfestation of underlying social norms that consistently direct girls to remain a virgin until marriage and to avoid initiating sex., while mixing messages for boys and ultimately engendered an expectation to dominate girls. Moreover, girls are likely to be blamed for the spread of HIV, as the community norms are restrictive for girls and permissive for boys, thus further discouraging girls from seeking sexual and reproductive health services, for fear of being shamed, blamed and punished, thus widening the taboo gap. These factors must be considered in sexual health education programmes, and it must be acknowledged that change will be gradual rather than immediate.

To combat the high rates of HIV infection in adolescent girls in Zambia, underlying social determinants of health must be addressed. Formal education and interventions that address food insecurity and enhance the economic stability of women and girls are crucial. More data are needed about current rates of child marriage and transactional sexual relationships. The recent rise in transactional relationships identified in South Africa^21^ could be a factor in Zambia as well and pose an important risk to adolescent health, although this requires further research to confirm (Conway-Smith, 2015). It will be crucial to address the underlying socioeconomic factors that often lead girls to pursue these types of relationships.

Key sources of influence for adolescent norms include peers, parents and the media (Kågesten et al., 2016) (Weber et al., 2019) (Cislaghi et al., 2021a, Cislaghi et al., 2021b) (Cohen et al., 2020) (Abdalla et al., 2020). We find evidence that strengthening peer support and parent-child interactions are important programmatic elements in addressing adolescent sexual and reproductive health knowledge and behaviours. Kågesten et al. (2016) similarly found in their systematic review of factors which shape gender attitudes in early adolescence that for programs to promote equitable gender attitudes, they need to shape interpersonal relationships with parents and peers as well as the wider social environment. Levy et al. (2020) found in their systematic review of the impact of programmes seeking to transform gender norms that the most effective, scalable and sustainable interventions tend to be multisectoral, and involve a wide array of stakeholders within local communities and multiple channels of action (Levy et al., 2020) (Hughes-d’Aeth, 2002). It will be crucial to incorporate health systems, churches, schools and peers, as well as parents and both male and female family members, in the process of addressing the harmful gender norms that contribute to the spread of HIV (Mmari and Magnani, 2003).

The concept of the taboo gap may be recognised in other situations, particularly where behaviours take place in private and are publicly sanctioned and discordant from commonly held attitudes. Discordance serves as a novel measure and harbinger for the presence of social norms, which in this case generated a taboo gap that impeded carseeking and increased risk for HIV, particularly among adolescent girls. Where community attitudes condemn premarital sex, yet premarital sex behaviour is common and thus discordant from attitudes, look for a taboo gap that prevents adolescents at high risk for HIV – or perhaps for other sexually transmitted diseases – from seeking and obtaining the services they need to practice safe sex. Programme success and adolescent lives, especially girls, depend on it.

## Author contributions

**Sophia Nesamoney**: Conceptualiztion, methodology, investigation, writing – original draft, visualization. **Iván Mejía-Guevara**: Formal analysis, data curation. **Ann Weber**: Writing – review and editing. **Beniamino Cislaghi**: Conceptualization, methodology, writing – review and editing. **Michael Mbizvo**: Writing – review and editing. **Gary Darmstadt**: Conceptualization, methodology, resources, writing – original draft, supervision, project administration, funding acquisition. All authors contributed intellectual content to paper drafts, reviewed and approved the final manuscript for publication.

## Funding

10.13039/100000865Bill and Melinda Gates Foundation grant #OPP1140262 to 10.13039/100005492Stanford University, Stanford, California, United States. The funder had no role in the writing of this manuscript.

## Figures and Tables

**figs1:**
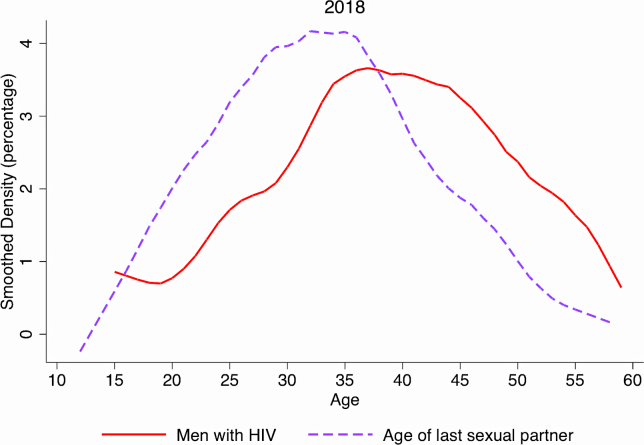


## Data Availability

All data used is publicly available.
