# Supplementary material for: Social normative origins of the taboo gap and implications for adolescent risk for HIV infection in Zambia
Source: Soc Sci Med. 2022 Nov;312:115391. doi: 10.1016/j.socscimed.2022.115391 (PMC9582197; doi:10.1016/j.socscimed.2022.115391)
Supplement: Multimedia component 1 [file mmc1.docx]

**Table S1.** Studies which inform the generation of norms related to the taboo gap in Zambia

|  | **Citation** | **Population Studied** | **Methods** | **Findings** |
| --- | --- | --- | --- | --- |
| ***Preventing HIV with young people: a case study from Zambia*** | Authors: Gordon G and Mwale V  Journal: Reproductive Health Matters  Date: 2006 | Eastern Province | Authors conducted a review of published literature and observations from field studies.  Research conducted in 2005-2006. | PEPFAR restricted schools to teaching abstinence-only sex education, through withholding funding if other forms of contraception were taught. Condom use could only be promoted for prostitutes, and it was forbidden to mention condoms in schools with students aged 10-14, despite data showing that many students were already sexually active at this age.  Zambia had high rates of HIV/AIDS in 2005-2006, as one in five youth were infected with the disease. The rates of HIV were six times higher in adolescent girls compared to adolescent boys. Of the sexually active adolescent girls in mentioned in the paper, 64% had experienced forced sex and 60% had already conceived children. |
| ***Community and health systems barriers and enablers to family planning and contraceptive services provision and use in Kabwe District, Zambia*** | Authors: Silumbwe A, Nkole T, Munakampe MN, Milford C, Cordero JP, Kriel Y, et al.  Journal: BMC Health Services Research  Date: 2018 | Central Province | Community members (114) and healthcare providers (19) were interviewed in focus group discussions. | Many women reported having to walk long distances to receive contraceptive care, which reduced their likelihood of care-seeking behaviour. Clinic staff were usually not trained in insertion of of intrauterine devices.  Another major complaint was the dismissive attitudes of healthcare providers, and many women felt unwelcome and/or were ridiculed when they did seek care. This effect was even more pronounced in unmarried adolescents, who reported being denied contraceptives and told that they should not be having sex in the first place.  There were also misconceptions around the effectiveness and safety of contraceptive methods. Many individuals reported being told that birth control pills would render their future children “lame” and give them “big heads.” They were also told that Mycrogynon use led to cancer. |
| ***Moving Beyond the ‘Male Perpetrator, Female Victim’ Discourse in Addressing Sex and Relationships for HIV Prevention: Peer Research in Eastern Zambia*** | Authors: Heslop J and Banda R  Journal: Reproductive Health Matters  Date: 2013 | Eastern Province | Through a partnership with the Young, Happy, Healthy, and Safe (YHHS) organization and the International HIV/AIDS Alliance, they recruited three male and three female peer researchers (aged 19-24). These peer researchers then interviewed 30 individuals aged 16-29 years. Of this sample, 12 were male and 18 were female, and 8 were married while 22 were not. The majority of study participants were not attending school.  Research was conducted in 2018. | Males were the dominant force in sexual and romantic relationships, and women/girls were expected to follow their lead. Participants thought that these attitudes were reinforced by the Bible and teachings at home/school. Girls were not allowed to refuse sex; consent was seen as a game for boys to win.  Initiation ceremonies appeared to be an important origin for these unbalanced gender norms. These initiation ceremonies occur at the onset of puberty, and for girls after the start of menstruation. Girls were given a special medication called lunkhanko that they were told would kill them if they engaged in premarital sex. During male initiation ceremonies, however, boys were given herbs to enhance sexual desire and were told that their masculinity was tied to their sexual frequency. |
| ***Adolescent HIV Disclosure in Zambia: Barriers, Facilitators, and Outcomes*** | Authors: Mburu G, Hodgson I, Kalibala S, Haamujompa C, Cataldo F, Lowenthal ED, and Ross D  Journal: Journal of the International AIDS Society  Date: 2014 | Adolescents in Zambia | Interviews were conducted with Zambian adolescents aged 10-19 who were HIV positive, as well as their parents and healthcare providers. The interviews were done as both semi-structured questionnaires and focus group discussions. | Due to restrictive norms around discussing sex and sexuality, many parents refrained from discussing HIV status with their children, so some of the adolescents had not been told that they had HIV. Of the adolescents who did know, some felt that they could share their status with others while others actively hid their status from friends and romantic partners due to stigma. |
| ***“When you have a high life, and you like sex, you will be afraid”: a qualitative evaluation of adolescents’ decision to test for HIV in Zambia and Kenya using the health belief model*** | Authors: Katirayi L, Akuno J, Kulukulu B, and Masaba R.  Journal: BMC Public Health  Date: 2021 | Adolescents in Lusaka, Zambia and Kisumu, Kenya | Interviews were conducted with Zambian and Kenyan adolescents aged 15-19. Some of the adolescents were HIV-positive while others did not know their HIV status. | Many adolescents who believed that they had a high probability of testing positive decided not to get tested for HIV because they were afraid of getting a positive HIV result. Many of these individuals saw HIV as a death sentence, so they said they would rather not know their status.  Some adolescents believed that suicide was a natural step after receiving an HIV-positive test; others said that their families would reject them if they became HIV positive.  Adolescents described barriers to testing in clinics; sometimes they were told that they were too young to get HIV tested or were unable to receive a test without a parent present. |
| ***“Bend a fish when the fish is not yet dry”: adolescent boys’ perceptions of sexual risk in Tanzania*** | Authors: Sommer M, Likindikoki S, Kaaya S.  Journal: Archives of Sexual Behavior  Date: 2015 | Adolescent boys in Tanzania | Interviews were conducted with 160 adolescent boys living in rural and urban Tanzania. | Many boys viewed puberty as justification for sex before marriage, stating that the sexual emotions that occur during male puberty cannot be resisted.  Boys also felt that their masculinity was tied to being able to be a provider of material goods and food for girls. Many of these boys mentioned providing these goods to girls in exchange for sex.  Boys also felt that using a condom reduced the pleasure of sex and some also believed that condoms had harmful bacteria or viruses in them. |
