# Supplementary material for: Social normative origins of the taboo gap and implications for adolescent risk for HIV infection in Zambia
Source: Soc Sci Med. 2022 Nov;312:115391. doi: 10.1016/j.socscimed.2022.115391 (PMC9582197; doi:10.1016/j.socscimed.2022.115391)
Supplement: Multimedia component 2 [file mmc2.docx]

**Table S2. Studies related to adolescent girls’ vulnerability to the taboo gap and HIV infection in Zambia**

|  | **Citation** | **Population Studied** | **Methods** | **Findings** |
| --- | --- | --- | --- | --- |
| ***Child Marriages in Zambia: A Study of Causal Factors in Selected Schools in Chipili District Zambia*** | Author: Kauseni BD  Journal: University of Zambia, Dissertation  Date: 2018 | Luapula Province | Teachers (n=10), parents (n=10), and students (n=30) in several schools across the Chipili district were interviewed. A comprehensive literature review on the subject was also conducted. | The rates of child marriage are the highest in rural regions and the lowest in the capital city. It is the highest In the Eastern Province (60% prevalence), followed by Luapala (50%), and the Northern province (48%). In the capital of Lusaka, the prevalence is 28%. According to the researcher, Zambia’s child marriage rates are among the highest in the world, as the average rate across the country is 42%.  There are several factors that shape the high incidence of child marriage, including polygamy, peer pressure, and community norms. Researchers found that parents often married their daughters young to satisfy elders in the community, but also as a way of protecting the daughters against HIV/AIDS and pregnancy out of wedlock. However, the researcher found that child marriage actually increases a young girl’s risk of acquiring HIV and of dying during childbirth. |
| ***An Examination of the Determinants of Sexual Behavior Among Young People Aged 15-24 Years in Zambia Using the 2005 Zambia Sexual Behavior Survey*** | Author: Mwale S  Journal: University of KwaZulu-Natal Durban, South Africa, Dissertation  Date: 2012 | Zambia at-large | Data from the 2005 Zambian Sexual Behavior Survey was used in this study. | The researcher found that there has been a delay in sexual debut since 2000 but that there has been minimal progress in implementing condom use. Condoms are often associated with populations that are sexually promiscuous, such as prostitutes, so most individuals avoid using them due to the stigma.  Condom promotion campaigns are important but still create a dynamic where women must depend on their male partners wanting/agreeing to use protection rather than them being able to have control over their own safety in sexual relationships. Condom use was more common in rural provinces than urban areas, suggesting that these campaigns were more aggressive and effective in the rural setting.  One of the most important findings was that the rates of HIV infection were 10% higher in married girls than in unmarried girls, which suggests that child marriage is not protective against HIV as was previously thought. |
| ***“When He Asks for Sex, You Will Never Refuse”: Transactional Sex and Adolescent Pregnancy in Zambia*** | Authors: Austrian K, Soler-Hampejsek E, Duby Z, Hewett PC  Journal: Studies in Family Planning  Date: 2019 | Zambia at-large | Qualitative interviews were conducted with adolescents in the Adolescent Girls Empowerment Program. An additional 1,853 longitudinal data samples were used, and these were from girls who had never been married or pregnant.  Research was conducted in 2013-2014. | One of the largest motivators for girls to engage in transactional sex with older men was food insecurity. Girls would engage in sex for as little as 0.80 USD regularly.  The study found that girls partaking in transactional sex were 37% more likely to become pregnant than those who were not. This was largely due to the imbalanced power dynamic in the relationship that prevented girls from negotiating for safe, protected sex. |
| ***Does Making Clinic-based Reproductive Health Services More Youth-friendly Increase Service Use by Adolescents? Evidence from Lusaka, Zambia*** | Authors: Mmari KN and Magnani RJ  Journal: Journal of Adolescent Health  Date: 2003 | Lusaka Province | Data was collected from 10 clinics in Lusaka, assessing indicators of youth-friendliness such as staff attitudes, staff ability to honor confidentiality, and the youth perception of the staff’s ability to meet their health needs. | Adults in the community believed that adolescents should not be seeking contraceptive care.  Adolescents believed that the nurses at the clinic were competent but did not feel comfortable engaging in discussions about sexual health with them. There were concerns about the clinicians passing judgment on the adolescents or denying them access to contraceptives. Many adolescents said that the clinicians could not be trusted to maintain patient confidentiality.  Several adolescents said that the experience could be made better by implementing a peer health counselor, which would make them more comfortable seeking care. |
| ***AIDS-Related Knowledge, Attitude, and Behavior Among Adolescents in Zambia*** | Authors: Mukuka L and Slonim-Nevo V  Journal: Ethnicity and Disease  Date: 2006 | Lusaka and Copperbelt Provinces | Youth were recruited from schools in these regions for 2-hour discussions about HIV/AIDS, in groups divided by gender. | Women are not allowed to seek sexual health treatment at the health clinics unless their partner is treated as well. This creates problems for young women, particularly those engaged in sex work, as there is a very low chance that their partners would agree to come to the clinic.  Although premarital sex is frowned upon in society, particularly for girls, the rates of sexual behaviour were very high for adolescents in this group.  In a separate focus group, the study authors interviewed adolescents living in the streets. Although these individuals had access to free condoms, they did not use them because they said that condoms interfere with sexual pleasure. Approximately 20% of the adolescents living in the streets had acquired a sexually transmitted disease in the past. |
| ***“My mother told me that I should not”: a qualitative study exploring the restrictions placed on adolescent girls living with HIV in Zambia*** | Authors: Mackworth-Young CR, Bond V, Wringe A, Konayuma K, Clay S, Chiiya C, et al.  Journal: Journal of the International AIDS Society  Date: 2017 | Adolescent girls in Lusaka, Zambia | Interviews were conducted with 24 adolescent girls in Lusaka, Zambia who were HIV positive. The girls also participated in four workshops. | Many of the girls felt that their parents were controlling and restrictive regarding their HIV status and that they were mostly told not to disclose their HIV status, not to have sex, and not to miss their treatment.  Many of these girls had not yet disclosed their HIV status to friends, and half of the girls had not yet disclosed their status to their families either. Many of these girls feared retaliation if they were to tell romantic partners, saying that if the relationship were to end, then their partner might get revenge by telling others their HIV status.  The researchers also found that the fear of unintentional disclosure sometimes also prevented girls from adhering to their HIV medications. Many of the girls also felt unsupported by community health workers but were very curious to ask questions of a doctor during the workshops when provided the opportunity. |
| ***Adolescent pregnancy and social norms in Zambia*** | Authors: Svanemyr J  Journal: Culture, Health, and Sexuality  Date: 2019 | Adolescents in Zambia | Interviews and focus group discussions were conducted with adolescent girls and boys in Zambia aged 13-18 years. | The researchers found conflicting gender norms that made it difficult for adolescents to know how they were supposed to behave. For instance, girls in the study mentioned that their peers would react negatively if they found that they had a boyfriend, but they also described feeling pressure from their peers to have a boyfriend. These girls also linked having a boyfriend with acquiring material goods such as snacks, cleaning items, and luxury goods. |
| ***Intersections of financial agency, gender dynamics, and HIV risk: a qualitative study with adolescent girls and young women in Zambia*** | Authors: Bermudez LG, Mulenga D, Musheke M, and Mathur S.  Journal: Global Public Health  Date: 2021 | Adolescent girls in Lusaka, Zambia | In-depth interviews were conducted with 30 adolescent girls aged 15-24 who lived in Kalingalinga, a low-income residential area in Lusaka, Zambia. | Girls described the importance of earning their own income, particularly when they reach age 18-20 years and want to have financial independence.  In the study, financial independence protected girls from high-risk sexual behaviours and prevented them from having to engage in transactional sex. It also gave them more decision-making power in relationships when men did not hold the economic power.  Girls in the study also described how women are primarily held responsible for preventing HIV in their relationships and that they are often the ones who insist on getting HIV tested and using condoms. |
| ***Interviewing adolescent girls about sexual and reproductive health: a qualitative study exploring how best to ask questions in structured follow-up interviews in a randomized controlled trial in Zambia*** | Authors: Isaksen K, Sandoy I, Zulu J, Melberg A, Kabombwe S, Mudenda M, et al.  Journal: BMC Reproductive Health  Date: 2022 | Adolescent girls in Zambia | Interviews and focus group discussions were conducted with adolescent girls in Zambia. | Many of the adolescent girls reported feeling shy and embarrassed when discussing sex, pregnancy, and abortion. Girls who had engaged in sexual behaviour reported feeling shame when asked questions about sex and many reported having been told by teachers and parents that sex before marriage was wrong and dangerous. |
