# Supplementary material for: Social normative origins of the taboo gap and implications for adolescent risk for HIV infection in Zambia
Source: Soc Sci Med. 2022 Nov;312:115391. doi: 10.1016/j.socscimed.2022.115391 (PMC9582197; doi:10.1016/j.socscimed.2022.115391)
Supplement: Multimedia component 3 [file mmc3.docx]

**Table S3.** Sexual and reproductive health education initiatives which inform approaches to address the taboo gap in Zambia

|  | **Citation** | **Population Studied** | **Methods** | **Findings** |
| --- | --- | --- | --- | --- |
| ***An Evaluation of the Effectiveness of a Peer Sexual Health Intervention Among Secondary-School Students in Zambia*** | Author: Agha S  Journal: AIDS Education and Prevention  Date: 2005 | Central, Lusaka, and Copperbelt Provinces | 913 students across secondary boarding schools were interviewed, and background information was collected on all students in each school. A skit about HIV prevention/safe sex was shown to the experimental group, while the control group learned about water purification. The knowledge and attitudes about sex were measured after the intervention. | Those in the experimental group had higher levels of knowledge about HIV than those in the control groups. These experimental students were also more likely to say that women could refuse sex and that condom use was important. |
| ***Do Peer Educators Make a Difference? An Evaluation of a Youth-Led HIV Prevention Model in Zambian Schools*** | Authors: Denison JA, Tsui S, Bratt J, Torpey K, Weaver MA, Kabaso M  Journal: Health Education Research  Date: 2012 | Zambian Central Province | Students in 8th and 9th grade at selected schools were interviewed. Intervention schools received lessons from trained peer health educators. Students were asked about their attitudes towards condoms, HIV, and safe sex, as well as their behaviours. They were also administered an HIV and reproductive health knowledge test. | Students in the intervention group had higher rates of correct answers on the HIV and reproductive health tests. They were also more likely to be aware of condom use as an effective method of preventing pregnancy and had greater confidence in refusing sex. However, the percentage of respondents reporting ever having had sex was the same across intervention and control schools, highlighting that the intervention may have changed attitudes but not necessarily behaviour. |
| ***Impact of Youth Peer Education Programs: Final Results from an FHI/YouthNet Study in Zambia*** | Svenson G, Burke H, Johnson L  Publication: Family Health International  Date: 2008 | Zambia at-large | Phase II of their study focused on peer health education programs in Zambia, using surveys and questionnaires. The surveys included both national (2005 Zambia Sexual Behavior Survey) and local clinic samples. | The rate of students receiving peer health education in Zambia at the time was 43%. Students seemed to benefit from peer health education, and students who received peer health education were more likely to use condoms and less likely to have sex with multiple partners. Peer health educators also played an important role in referring students with at-risk sexual histories to clinics, where they could receive medical care. |
| ***Evaluation of HIV/AIDS peer education projects in Zambia*** | Hughes-d’Aeth A  Publication: Evaluation and Program Planning  Date: 2002 | Lusaka, Eastern Province, and Copperbelt | The study authors observed the activities of four NGOs in Zambia working to implement HIV/AIDS education. They conducted interviews with students. | They concluded that a multisectoral approach was key. Integrated psychological services and information about income generation was important, as was implementing solutions through the cultural context of urbanisation, sanitation, and poverty. |
| ***Let us fight and support one another: adolescent girls and young women on the contributors and solutions to HIV risk in Zambia*** | Butts S, Parmley L, Alcaide M, Rodriguez V, Kayukwa A, Chitalu N, Weiss S, Jones D | Lusaka, Copperbelt, and Southern Provinces | The study authors spoke to 225 adolescent girls and women from the Zambian provinces with the highest HIV prevalence. Focus group discussions were conducted. | They concluded that it was important to incorporate multiple types of media to convey information about sexual health. Girls also said that there should be a greater emphasis on privacy and confidentiality in sexual health clinics and that there should be more grave consequences for those who breached this. They also said that peer educators should be trained for situations where a student may confide in them about rape or assault. |
| ***Feasibility, Acceptability, and Preliminary Efficacy of Tikambisane (‘Let’s Talk to Eachother’): A Pilot Support Group Intervention for Adolescent Girls Living with HIV in Zambia*** | Stangl AL, Mwale M, Sebany M, Mackworth-Young CR, Chiiya C, Chonta M, et al.  Publication: Journal of the International Providers of AIDS Care  Date: 2021 | Chipata and Kanyama, Zambia | In-depth interviews were conducted with 21 adolescent girls living in Chipata and Kanyama aged 15-19 years. They were testing an intervention of a 6-session curriculum called Tikambisane for girls living with HIV. The curriculum included discussions about topics such as grief, relationships, anti-retroviral therapy (ART), and disclosure, as well as role playing sessions. | Participants described how having peer co-facilitators helped them to feel more comfortable and fostered unity in the group. After the intervention, the subjects were excited to learn more about HIV and ART and engage in additional discussions around relationships and health. The girls also said they appreciated being able to form support networks with other HIV-positive girls since they did not have those networks in place prior to joining this intervention. |
| ***A review of interventions addressing structural drivers of adolescents’ sexual and reproductive health vulnerability in sub-Saharan Africa: implications for sexual health programming*** | Wamoyi J, Mshana G, Mongi A, Neke N, Kapiga S, and Changalucha J.  Publication: BMC Reproductive Health  Date: 2014 | Sub-Saharan Africa | Summary of existing interventions in sub-Saharan Africa | Girls Power Initiative: Girls became economically empowered through skills training activities, which reduced the likelihood that they would pursue transactional sex.  Families Matter! Initiative: Focused on improving the parent-child dynamic as it related to discussions about sex and safe sex. Parents received five 3-hour workshops about how to help their children prevent HIV. This initiative proved effective in improving the parent-child dynamic and increasing the frequency of parent-child communication about sexuality and HIV risk reduction. |
| ***Application of community dialogue approach to prevent adolescent pregnancy, early marriage, and school dropout in Zambia: a case study*** | Zulu IZ, Zulu JM, Svanemyr J, Michelo C, Mutale W, Sandoy IF.  Publication: BMC Reproductive Health  Date: 2022 | Zambia | Dialogues about sexual and reproductive health were implemented in communities, and community attitudes towards these interventions were assessed. | Parents were initially afraid of discussing sex with their children, but they said that the lessons they learned through these interventions enabled them to communicate better with their children and helped them to understand the specific challenges that their children are facing. They also said that they became more aware of the importance of education and female empowerment and several parents decided to delay marriage for their daughters. However, some parents did feel angry that contraception was taught in the workshops and felt that this was inappropriate. |
